# Rush Medical College

**Published:** 1867-02

**Authors:** 


Rush Medical College.—The closing exercises of the Twenty-Fourth Annual Session of this institution were held in the lecture-room of the college on the evening of January 30.

The address to the graduating class was delivered by Prof. E. Ingalls, and will be given in our next issue. It was a most excellent production, replete with valuable advice, which not only students but also physicians long in practice, would do well to follow. The degree of doctor of medicine was conferred upon 72 members of the class, by Prof. Blaney, recently elected to the presidency of the college, made vacant by the death of Prof. Brainard. The honorary degree was conferred upon Dr. David Prince, of Jacksonville, and Dr. Ezra S. Carr, of Madison, Wisconsin. We have been informed by the Secretary of the College that the class in attendance was never before so large as during the past session.

Prof. M. Gunn, M.D., of the Medical Department of the University of Michigan, has accepted an appointment to the Chair of Surgery made vacant by the death of Prof. Brainard.

A professorship of Surgical Anatomy and Military Surgery has been created, to which Prof. Edwin Powell has been elected.

